# Draft genome of *Conoideocrella luteorostrata* ARSEF 14590 (Clavicipitaceae), an entomopathogenic fungus with a wealth of biosynthetic and biocontrol potential

**DOI:** 10.1128/mra.01273-24

**Published:** 2025-07-23

**Authors:** Brian Lovett, Jason E. Stajich, Hana Barrett, Lindsay R. Kasson, Daniel G. Panaccione, Cecilia A. Reiter, Jessica L. Fuss, Gregory Biddle, Matt T. Kasson

**Affiliations:** 1 Department of Entomology, Cornell University 171521, Ithaca, New York, USA; 2 Department of Microbiology and Plant Pathology, University of California-Riverside 207030, Riverside, California, USA; 3 Plant Pathology & Plant-Microbe Biology Section, School of Integrative Plant Science, Cornell University 5922, Ithaca, New York, USA; 4 School of Medicine, West Virginia University 12355, Morgantown, West Virginia, USA; 5 School of Natural Resources and the Environment, West Virginia University 717118 , Morgantown, West Virginia, USA; University of Maryland School of Medicine, Baltimore, Maryland, USA

**Keywords:** *Torrubiella luteorostrata*, *Paecilomyces cinnamomeus*, insect pathogen, *Fiorinia externa*, elongate hemlock scale

## Abstract

The fungus *Conoideocrella luteorostrata* is a recently discovered pathogen of invasive elongate hemlock scale insects (EHS; *Fiorinia externa*) in Christmas tree farms in the eastern U.S. Here, we report a scaffold-level genome and assembly along with an initial survey of biosynthetic gene clusters for strain ARSEF 14590 from EHS.

## ANNOUNCEMENT

**Fig 1:**
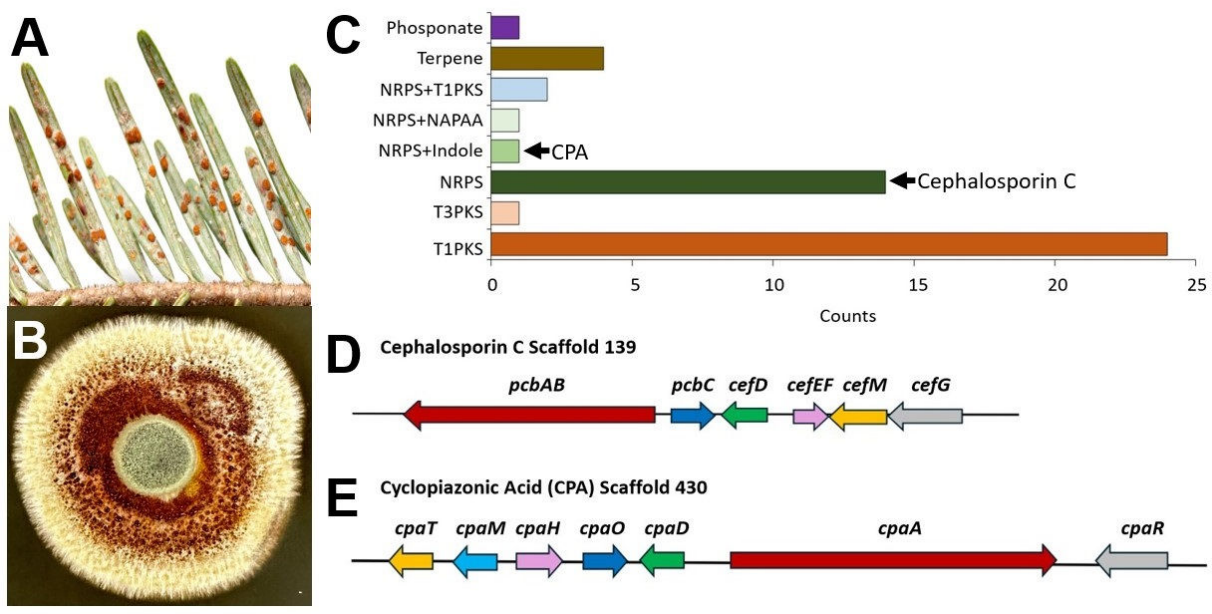
*Conoideocrella luteorostrata* (**A**) *in situ* sporulating atop infected *Fiorinia externa* nymphs (courtesy of Dr. Matt Bertone, NCSU, reproduced with permission) and (**B**) *in vitro* (strain ARSEF 14590) after 2 weeks growth on potato dextrose agar (courtesy of Matt Kasson). (C) AntiSMASH predicted biosynthetic gene clusters (BGCs) for ARSEF 14590 across all classes. Intact BCGs for both cephalosporin C (D) and cyclopiazonic acid (**E**) were found in ARSEF 14590. Cephalosporin C BGC includes: *pcbAB*, acv synthetase; *pcbC*, isopenicillin N synthase; *cefD*, isopenicillin N epimerase; *cefEF*, cephalosporin biosynthesis expandase/hydroxylase; *cefM*, transporter (multi-drug resistance homolog); and *cefG*, isopenicillin N-CoA synthetase. Cyclopiazonic Acid BGC includes: *cpaT*, MFS transporter; cpaM, N-methyltransferase, cpaH, Cytochrome P450 monooxygenase; cpaO, monoamine oxidase; cpaD, Tryptophan dimethylallyltransferase; cpaA, a multifunctional CPA synthase; and cpaR, transcription factor.

Our recent discovery of epizootics of the entomopathogenic fungus *Conoideocrella luteorostrata* (*CL*) on elongate hemlock scale insects (EHS; *Fiorinia externa*) in Christmas tree orchards in North Carolina (1) and by others elsewhere in the United States (2) has generated interest regarding its use as a biological control agent (Fig. 1). *CL* was reported from various Hemiptera across the southeastern United States in the early 1900s (1), yet it remains unclear if earlier accounts were actually caused by *Aschersonia* (1, 3, 4), given its overlapping morphology and host range. >230 Clavicipitaceae genomes have been sequenced to date (5), but a majority belong to just five of >50 recognized genera (6, 7) including *Claviceps*, *Epichloë*, *Ustilaginoidea*, *Metarhizium*, and *Aschersonia* (8–15). Genome sequencing is urgently needed for the remaining genera, including *Conoideocrella,* given ongoing taxonomic conflicts (1, 16) and recent discoveries (17, 18).

A mycelial culture of the recently characterized strain ARSEF 14590 (1) isolated from an infected EHS crawler in Ashe County, North Carolina (Table 1) in 2020 was grown out for ~2 weeks on potato dextrose agar at 22°C. Genomic DNA was extracted from fungal tissue with a Qiagen DNeasy PowerSoil Pro Kit. DNA sequencing libraries were prepared with sparQ DNA fragment and library kit (Quanta Bio, Beverly, MA), which uses enzymatic shearing to fragment and sizing, and DNA quantitation was performed on an Agilent 2100 Bioanalyzer System and High Sensitivity DNA Kit using the manufacturer’s protocols. An Illumina NextSeq 1000 (Marshall University Genomics Core Facility, Huntington, WV) generated 8.668M 2 × 150 bp paired sequence reads or 2.6 Gb from *CL* ARSEF 14590. The assembled scaffold-level (*n* = 864) genome for *CL* strain ARSEF 14590 was 47.39 Mbp (coverage, 51.6 x; *N*_50_, 111.30 kb; *L*_50_, 126; G + C content, 49%). The assembly was cleaned of vector contamination and redundant contigs were cleaned as previously described (19) using SPAdes v3.15.2 (20) run within AAFTF (v0.4.1; 21) with the trim and filter steps to quality control the reads with fastp (v0.23.2; 22) and BBMap (v39.19; 23) to remove phiX and adaptor sequence, before running the assembly step using defaults set in AAFTF. This is followed by vectrim, sourpurge with phylum = Ascomycota, and rmdup steps to remove contaminating contigs with default parameters. Assemblies were subjected to five rounds of polishing with Pilon (v1.24; 24) with Illumina reads. Genome annotation was performed with funannotate (v1.8.15; 25) utilizing alignment of proteins in UniProt and BUSCO with sordariomycetes_odb10 for training, and tRNA genes were predicted using tRNAscan-SE v2.0.9 (26). BUSCO v5.4.4 (27), using the ascomycota_odb10 data set (28), identified 1,696/1,706 (99.5%) complete, 1,684 (98.7%) single copy, 14 (0.8%) duplicated, and 2 (0.1%) fragmented markers. Default parameters were used or when specified, available in the pipeline code, parameters, and logfiles archived in Github and Zenodo (29). The final genome annotation included a total of 12,844 protein-coding genes and 110 tRNAs. AntiSMASH v5.0 (30) predicted 48 biosynthetic gene clusters (BGCs) for ARSEF 14590 using “strict” parameters*,* including 10 NRPS and 24 T1PKS BGCs (Table 1). Intact NRPS-containing BGCs for cephalosporin C, an important β-lactam antibiotic (31), and cyclopiazonic acid, a fungal neurotoxin chemically related to ergoline alkaloids (32) (Fig. 1), highlights just some of the BGCs whose natural products may serve important roles in *CL*-EHS interactions with additional potential applications in medicine and agriculture.

**TABLE 1:** Summary of biosynthetic gene cluster data for *Conoideocrella luteorostrata* ARSEF 14590 and other Clavicipitaceae reference genomes

Species	Strain no.	Genome assembly	Size (Mb)	NRPS	NRPS_T1PKS	NRPS_Other^*b*^	T1PKS	Terpene	Indole	NAPAA	Isocyanide	Other^*c*^	Total BCGs
*Aschersonia sp.*	MBC 622	GCA_030782805.1	32.68	13	4	2	11	5	0	1	1	0	37
*Aschersonia sp.*	MBC 508	GCA_030780645.1	31.39	4	1	1	7	2	0	0	2	0	17
*Aschersonia sp.*	MBC 710	GCA_030783285.1	31.32	5	1	1	9	3	0	0	2	0	21
*Aschersonia sp.*	MBC 593	GCA_030780945.1	29.97	2	1	1	9	4	0	0	2	1	20
*Aschersonia sp.*	MBC 735	GCA_030783925.1	26.99	5	0	1	11	5	1	1	2	3	29
*Aschersonia sp.*	MBC 819	GCA_030785145.1	26.95	4	1	0	11	5	2	1	2	3	29
*Aschersonia sp.*	MBC 612	GCA_030781705.1	26.91	5	1	0	11	6	2	1	2	3	31
*Aschersonia sp.*	MBC 877	GCA_030787035.1	26.89	5	1	1	7	6	1	1	2	3	27
*Aschersonia sp.*	MBC 610	GCA_030781625.1	26.82	5	1	1	9	6	1	0	2	4	29
*Balansia obtecta*	B249	GCA_000709145.1	30.15	1	2	1	4	2	0	1	0	0	11
*Claviceps arundinis*	CCC 1102	GCA_018360175.1	30.33	9	0	0	6	4	1	1	0	0	21
*Claviceps bavariensis*	CCC434	GCA_004016155.1	30.49	7	0	0	8	4	1	1	0	0	21
*Claviceps fusiformis*	PRL 1980	GCA_000223055.1	52.59	3	0	0	6	2	2	1	0	0	14
*Claviceps monticola*	CCC 1483	GCA_018360055.1	27.64	7	0	0	6	3	2	1	0	0	19
*Claviceps occidentalis*	PRL1580	GCA_004016105.1	28.57	7	0	0	7	2	1	1	0	0	18
*Claviceps paspali*	ILB388	GCA_013435705.1	29.21	4	2	0	4	4	2	1	0	0	17
*Claviceps pazoutovae*	CCC 1485	GCA_018360065.1	27.8	7	0	0	7	2	1	1	0	0	18
*Claviceps purpurea*	LM72	GCA_029405325.1	34.19	6	0	1	8	3	2	0	0	0	20
*Claviceps quebecensis*	136	GCA_004016085.1	35.88	6	0	1	6	2	1	0	0	1	17
*Conoideocrella luteorostrata*	ARSEF 14590^*a*^	GCA_032433595.1	47.39	14^*d*^	2	2	24^*d*^	4	0	0	0	2	48
*Epichloe amarillans*	NFE708	GCA_024072835.1	38.12	10	0	1	6	4	0	1	1	2	25
*Epichloe baconii*	E357	GCA_023650635.1	39.19	5	0	0	10	3	1	1	1	1	22
*Epichloe brachyelytri*	E1124	GCA_023650705.1	44.64	6	0	0	10	3	0	0	1	0	20
*Epichloe elymi*	Nfe728	GCA_023658475.1	34.12	7	2	0	6	4	1	1	1	2	24
*Epichloe festucae*	Fl1	GCA_003814445.1	34.97	8	0	2	8	4	0	1	0	2	26
*Epichloe glyceriae*	E2772	GCA_023658535.1	42.97	3	0	0	7	1	1	0	1	1	14
*Epichloe scottii*	TT-2021a	GCA_021950295.1	37.25	9	1	0	5	3	0	0	1	2	21
*Metarhizium acridum*	ARSEF 324	GCA_019434415.1	44.71	12	3	1	9	5	2	0	1	0	33
*Metarhizium album*	ARSEF 1941	GCA_000804445.1	30.45	8	3	1	9	3	0	1	1	1	27
*Metarhizium anisopliae*	MEAPA 0093	GCA_039654215.1	39.55	11	7	2	17	2	2	1	1	3	46
*Metarhizium brunneum*	4556	GCA_013426205.1	37.77	8	7	1	19	3	1	1	1	2	43
*Metarhizium guizhouense*	ARSEF 977	GCA_000814955.1	43.47	14	9	1	16	6	3	1	2	2	54
*Metarhizium humberi*	ESALQ1638	GCA_020102295.1	38.59	12	8	1	21	3	3	1	1	3	53
*Metarhizium pinghaense*	M-1000	GCA_041379975.1	44.29	11	6	1	23	4	4	1	2	4	56
*Metarhizium rileyi*	MrS1GZL-1	GCA_031753515.1	34.73	9	3	0	9	2	1	1	0	1	26
*Metarhizium robertsii*	ARSEF 23	GCA_000187425.2	41.66	10	6	1	19	6	3	1	1	4	51
*Metarhizium sp.*	BCC 4849	GCA_037953715.1	37.98	12	6	2	18	2	2	0	1	2	45
*Moelleriella libera*	RCEF 2490	GCA_001636675.1	30.87	4	1	0	6	5	0	1	0	3	20
*Periglandula ipomoeae*	IasaF13	GCA_000222875.2	35.3	10	0	0	7	1	0	0	0	0	18
*Pochonia chlamydosporia*	170	GCA_001653235.2	44.22	10	3	0	14	8	0	1	0	1	37
		Average BGCs	35.23	7	2	1	10	4	1	1	1	1	28

^*a*^ Strain ARSEF 14590 was isolated from a mycosed elongate hemlock scale insect collected at NC State University's Upper Mountain Research Station in Ashe County, North Carolina, USA (36.400327°N–-81.305294°W) on 31 August 2020.

^*b*^ NRPS+(Other) includes NRPS + Terpene, NRPS + Indole, NRPS + NAPAA, and Isocyanide + NRP.

^*c*^ Other category includes T1PKS + terpene, T1PKS + Indole, Trans-AT PKS, T3PKS, Terpene + Indole, Betalactone, fungal-RIPP, and Phosponate.

^*d*^ The foundational NRPS or T1PKS in each *C. luteorostrata* cluster was manually confirmed to be free or frame shifts or premature stop codons.

## Data Availability

This whole-genome shotgun project has been deposited at DDBJ/ENA/GenBank under the accession number JASWJB010000000 and the assembly under accession number GCA_032433595.1. Sequence reads were deposited under SRA project accession number SRX20698148, BioProject accession number PRJNA980380, and BioSample accession number SAMN35627742.
